# Glycemic control in gestational diabetes and impact on biomarkers in women and infants

**DOI:** 10.1038/s41390-022-02459-0

**Published:** 2023-01-17

**Authors:** Olivia J. Hofer, Jane Alsweiler, Thach Tran, Caroline A. Crowther

**Affiliations:** 1 Faculty of Medical and Health Sciences, University of Auckland, Auckland, 1023 New Zealand; 2 Liggins Institute, University of Auckland, Auckland, 1023 New Zealand; 3 Department of Paediatrics: Child and Youth Health, University of Auckland, Auckland, 1023 New Zealand; 4 School of Biomedical Engineering, University of Technology Sydney, Sydney, NSW 2007 Australia

## Abstract

**Background:**

Gestational diabetes mellitus (GDM) is linked to the dysregulation of inflammatory markers in women with GDM compared to women without. It is unclear whether the intensity of glycemic control influences these biomarkers. We aimed to assess whether different glycemic targets for women with GDM and compliance influence maternal and infant biomarkers.

**Methods:**

Maternity hospitals caring for women with GDM were randomized in the TARGET Trial to tight or less tight glycemic targets. Maternal blood was collected at study entry, 36 weeks’ gestation, and 6 months postpartum, and cord plasma after birth. We assessed compliance to targets and concentrations of maternal serum and infant biomarkers.

**Results:**

Eighty-two women and infants were included in the study. Concentrations of maternal and infant biomarkers did not differ between women assigned to tighter and less tight glycemic targets; however, concentrations were altered in maternal serum leptin and CRP and infant cord C-peptide, leptin, and IGF in women who complied with tighter targets.

**Conclusions:**

Use of tighter glycemic targets in women with GDM does not change the concentrations of maternal and infant biomarkers compared to less tight targets. However, when compliance is achieved to tighter targets, maternal and infant biomarkers are altered.

**Impact:**

- The use of tighter glycemic targets in gestational diabetes does not result in changes to maternal or cord plasma biomarkers. However, for women who complied with tighter targets, maternal serum leptin and CRP and infant cord C-peptide, leptin and IGF were altered compared with women who complied with the use of the less tight targets.
- This article adds to the current evidence base regarding the impact of gestational diabetes on maternal and infant biomarkers.
- This article highlights the need for further research to assess enablers to meet the tighter target recommendations and to assess the impact on relevant biomarkers.

## Introduction

Gestational diabetes mellitus (GDM) is characterized by any degree of glucose intolerance with onset or recognition during pregnancy.^1^ The pathophysiology of GDM has been linked to dysregulation of inflammatory markers that interfere with the action of insulin,^2^ heightening the state of insulin resistance typically seen in pregnancy.^3^ There are known differences in the profile of cytokines and adipokines in women with GDM compared to pregnant women without GDM. In women with GDM, the proinflammatory cytokines tumor necrosis factor-α^4^ and C-reactive protein (CRP),^5–7^ leptin,^4^ and triglycerides^8^ are significantly elevated; some throughout pregnancy, others only in the third trimester, while adiponectin concentrations appear to be significantly lower contributing to the higher leptin/adiponectin ratio.^9^ The extent of the reduction in adiponectin during pregnancy results in fetal overgrowth and correlates with the risk of having a large-for-gestational-age (LGA) infant.^3,9^ Whether infants born to women with GDM have an altered cord adipokine and cytokine profile remains unclear, with data limited to small case–control studies with conflicting results.^10–15^ Two large longitudinal cohort studies of pregnant women with normoglycemia, the Rhea study^16^ and Project Viva,^17^ have shown that cord adipokines concentrations are predictive of infant birth weight and growth trajectories. Understanding these markers in infants born to women with GDM may be important in predicting their risk of childhood obesity and metabolic syndrome.

The degree to which maternal glucose control affects these biomarkers markers is uncertain. A nested study with the ACHOIS randomized trial suggested that cord adiponectin and leptin concentrations in infants born to women with GDM are influenced by treatment that included dietary advice and pharmacotherapy where necessary.^18^ A recent small, randomized trial (*n* = 41) that allocated women with GDM to either tight (fasting plasma glucose (FPG) < 5.1 mmol/L) or less tight (FPG < 5.3 mmol/L) glycemic targets reported higher cord plasma leptin concentrations and leptin/adiponectin ratios in infants of women in the less tight group.^19^ However, no difference was found in C-peptide or adiponectin concentrations between the two glycemic targets groups.^19^

A randomized trial that compared women with GDM assigned to biweekly prenatal clinic care with women with GDM assigned to additional daily feedback on their compliance to glycemic control found that while 66% of women receiving routine care were able to achieve their glycemic targets, this increased to 84% for women in the daily feedback group.^20^ Furthermore, a survey in women with GDM on their experience of achieving their recommended glycemic targets found that 62% described achieving fasting targets as the most difficult, and 62% of women were always or frequently hungry.^21^ When considering the literature surrounding the use of tighter glycemic targets and changes in biomarkers, it is important to consider whether adherence to tighter and less tight targets affects maternal and infant biomarkers.

Our study aimed to assess whether different intensities of glycemic targets for pregnant women with GDM influence the maternal cardiometabolic markers of triglycerides, cholesterol, CRP, leptin, and adiponectin, and infant cardiometabolic, growth, and inflammatory markers of cord plasma C-peptide, leptin, adiponectin, and insulin-like growth factor (IGF). In addition, we assessed whether achieving compliance to different intensities of glycemic control for pregnant women with GDM influenced these cardiometabolic, growth, and inflammatory markers.

## Methods

This study was nested within the TARGET Trial, a stepped-wedge, cluster, randomized controlled trial, the methodology of which has been described previously^22^ and the results have been published.^23^ Briefly, the TARGET Trial randomized 10 participating hospitals to a timing change from the use of less tight glycemic targets (FPG < 5.5 mmol/L; 1 h postprandial <8.0 mmol/L; 2 h postprandial <7.0 mmol/L) to the use of tighter glycemic targets (FPG ≤ 5.0 mmol/L; 1 h postprandial ≤7.4 mmol/L; 2 h postprandial ≤6.7 mmol/L) for treating women with gestational diabetes. Women who were recruited from four hospitals participating in the trial that were able to collect blood at the four study time points were considered relevant for this study. At these hospitals, 328 women enrolled in the trial were invited to participate in the biomarker study if they had a singleton pregnancy, gave informed consent to have a blood sample taken at trial entry, 36 weeks gestation, 6 months after the birth, and for cord plasma to be collected after the birth. Glucometer data were collected from participating women to determine whether compliance with at least 80% of fasting, postprandial, or both fasting and postprandial targets was achieved. The TARGET Trial was registered with the Australian New Zealand Clinical Trials Registry—ACTRN 12615000282583. Human ethics approval was granted by the Northern A Health and Disability Ethics Committee in New Zealand (14/NTA/163/AMO1). All participants provided informed consent for participation in this nested study.

Maternal blood was collected at entry to the study, 36 weeks, and 6 months postpartum into lithium heparin-coated tubes (BD vacutainer 367526) and was centrifuged at 1300 × *g* at 4 °C for 10 min. Plasma was collected, aliquoted, and stored at −80 °C until further analysis. Cord plasma samples were collected at birth and handled using the previously described procedure. At the time of analysis, samples were thawed on ice and processed according to the manufacturer’s protocol. Samples were assayed blind to glycemic targets and measured in duplicate. Cholesterol, triglycerides, and CRP concentrations were measured using Cobas Autoanalyzer C311.^24^ IGF-1 was measured using ELISA Abcam Simple Step.^25^ Adiponectin and leptin concentrations were analyzed with Magnetic Luminex^®^ Assay.^26^ C-peptide concentration was measured using Cobas autoanalyzer E411.^27^ Appropriate calibrators and quality controls were performed at the time of analysis.

### Statistical analysis

Baseline data are presented descriptively as median (interquartile range) and number (%) for continuous and categorical variables, respectively. The analyses were carried out using the intention-to-treat approach in which enrolled participants were analyzed according to the treatment targets their hospital was randomized to and when their diagnosis of GDM was made. We used generalized linear mixed-effects models (GLMM) to evaluate the main treatment effect, with a random effect for hospital groups and the participants, and fixed effects for the intervention implementation and time. Time was estimated in months between the date the assigned targets were initiated and the date a woman enrolled. Time was included in the model to account for secular trends over time as the study design induces an association between time and outcome of interest, and failure to include time into the model might bias the effect size estimates.^28^ The primary analyses of biomarkers made adjustment for gestational age (GA) at OGTT and baseline values. We conducted a predefined exploratory analysis that further adjusted for the baseline predictors of BMI, ethnicity, and history of diabetes that showed evidence of important imbalance between the glycemic target groups.

The analyses were conducted to examine the difference in means of biomarkers between the tighter targets group compared to the less tight targets group using their logarithm-transformed data. The tighter target group included data from women with GDM recruited in the tighter target period, while the less tight target group used data from women recruited in the less tight target period. We reported differences in the percentages between the means of the tighter and the less tight target group (95% confidence interval), which was derived from back logarithm transforming the estimated coefficients estimated from the GLMM (i.e., ~(exponential of the estimated coefficient − 1) × 100%). Compliance was defined as achieving ≥80% of either fasting, postprandial or both fasting and postprandial targets and analyses were performed as above for these subgroups. A *P* value of <0.05 was considered statistically significant. Given the exploratory nature of the study, no adjustment was made for multiple comparisons. All analyses were conducted using the SAS software 9.4 (SAS Institute, Inc., Cary, NC).

## Results

### Sample characteristics

**Fig. 1:**
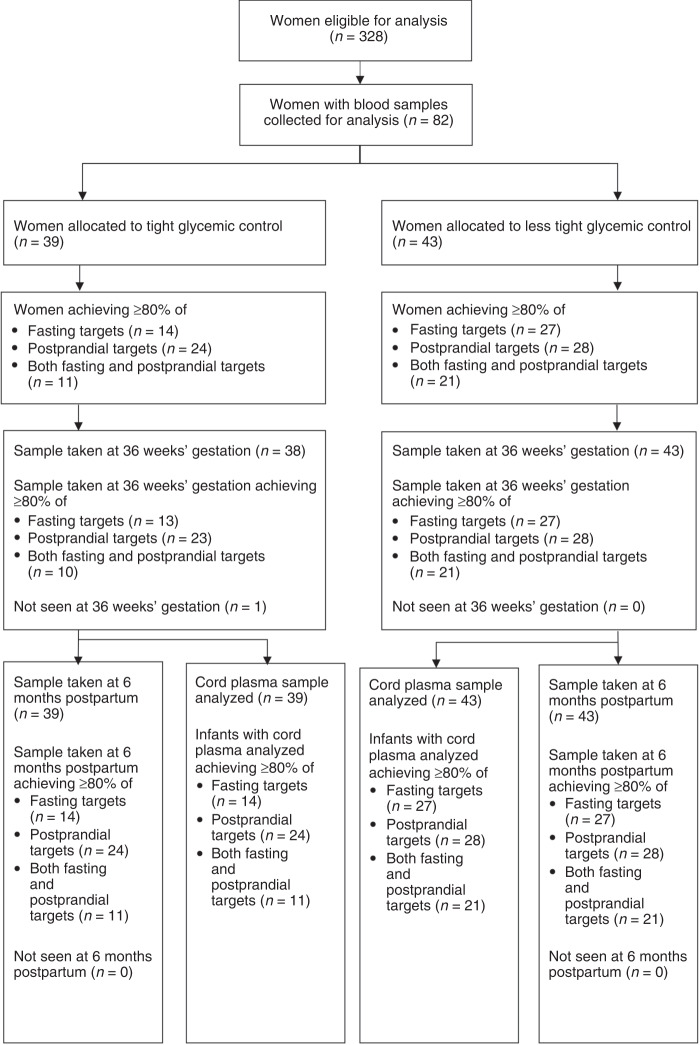
Women and infants in the study cohort. Study profile of blood samples from women and infants by tight or less tight glycemic control.

A total of 82 of 328 (25%) women had blood samples taken at trial entry, 36 weeks’ gestation, and 6 months after the birth and 82 infants had blood taken from the umbilical cord after birth and were included in this cohort study (Fig. 1). Thirty-nine women used the tighter glycemic targets, and 43 women the less tight glycemic targets. The baseline characteristics of women at study entry were similar between the glycemic target groups, including for gestational age at study entry and parity (Table 1). More women had a previous history of GDM in the tighter target group compared with women in the less tight group (10 (25.6%) vs. 3 (7.0%), *P* = 0.02). There were no differences between the two glycemic target groups for median maternal cardiometabolic markers at trial entry (Table 1).

**Table 1:** Baseline characteristics of the women.

Characteristics	All participants			≥80% of fasting targets			≥80% of postprandial targets			≥80% of both fasting and postprandial targets		
	Tighter targets (*n* = 39)	Less tight targets (*n* = 43)	*P* value	Tighter targets (*n* = 14)	Less tight targets (*n* = 27)	*P* value	Tighter targets (*n* = 24)	Less tight targets (*n* = 28)	*P* value	Tighter targets (*n* = 11)	Less tight targets (*n* = 21)	*P* value
Primiparous	17 (43.6)	21 (48.8)	0.63	7 (50.0)	17 (63.0)	0.42	10 (41.7)	17 (60.7)	0.17	6 (54.5)	15 (71.4)	0.34
BMI (kg/m^2^)^a^	28.3 (26.0–34.6)	30.6 (26.8–35.5)	0.32	26.9 (25.5–34.6)	29.5 (25.8–32.4)	0.49	27.5 (25.9–34.4)	29.5 (26.7–32.8)	0.50	26.4 (25.5–34.6)	29.3 (25.9–30.7)	0.59
BMI category			0.47			0.43			0.46			0.48
Normal	6 (15.4)	5 (11.6)		3 (21.4)	5 (18.5)		3 (12.5)	3 (10.7)		2 (18.2)	3 (14.3)	
Overweight	17 (43.6)	14 (32.6)		7 (50.0)	9 (33.3)		12 (50.0)	12 (42.9)		6 (54.5)	9 (42.9)	
Obese	16 (41.0)	24 (55.8)		4 (28.6)	13 (48.1)		9 (37.5)	13 (46.4)		3 (27.3)	9 (42.9)	
Ethnicity			0.32			0.50			0.36			0.57
NZ European	21 (53.8)	22 (51.2)		8 (57.1)	12 (44.4)		14 (58.3)	16 (57.1)		6 (54.5)	11 (52.4)	
Pacifica	1 (2.6)	0 (0.0)		0 (0.0)	0 (0.0)		2 (8.3)	0 (0.0)		0 (0.0)	0 (0.0)	
Māori	3 (7.7)	0 (0.0)		0 (0.0)	0 (0.0)		1 (4.2)	0 (0.0)		0 (0.0)	0 (0.0)	
Asian	13 (33.3)	17 (39.5)		5 (35.7)	12 (44.4)		6 (25.0)	9 (32.1)		4 (36.4)	7 (33.3)	
Other	1 (2.6)	4 (9.3)		1 (7.1)	3 (11.1)		1 (4.2)	3 (10.7)		1 (9.1)	3 (14.3)	
Previous gestational diabetes^b^	10/22 (45.5)	3/22 (13.6)	0.05	3/7 (42.9)	1/10 (10.0)	0.12	5/14 (35.7)	2/11 (18.2)	0.33	1/5 (20.0)	1/6 (16.7)	0.89
Gestational age at entry (weeks.days)^a^	27.6 (26.4–28.6)	27.6 (26.2–28.5)	0.46	28.3 (26.5–29.2)	27.6 (27.0–28.5)	0.35	28.2 (26.9–28.8)	27.5 (25.9–28.5)	0.18	28.2 (26.5–29.2)	28.0 (27.0–29.2)	0.46
OGTT: fasting result (mmol/L)^a^	4.9 (4.5–5.7)	5.0 (4.5–5.6)	0.84	4.6 (4.5–5.5)	4.7 (4.4–5.2)	0.87	5.0 (4.5–5.6)	4.8 (4.4–5.3)	0.35	4.9 (4.5–5.8)	4.5 (4.3–4.9)	0.10
OGTT 2 h result (mmol/L)^a^	9.2 (9.0–9.8)	9.3 (8.7–10.7)	0.25	9.4 (9.0–9.6)	9.4 (9.1–10.1)	0.54	9.2 (8.9–9.5)	9.4 (8.6–10.1)	0.45	9.2 (8.8–9.5)	9.4 (9.1–10.0)	0.25
Systolic blood pressure (mmHg)^a^	110.0 (100.0–118.0)	110.0 (102.0–120.0)	0.84	108.0 (100.0–118.0)	105.0 (100.0–116.0)	0.65	110.0 (100.0–115.0)	110.0 (102.0–120.0)	0.39	105.0 (95.0–118.0)	108.0 (100.0–120.0)	0.65
Diastolic blood pressure (mmHg)^a^	65.0 (56.0–74.0)	70.0 (60.0–80.0)	0.23	68.0 (52.0–74.0)	64.0 (60.0–80.0)	0.68	62.0 (53.5–70.0)	70.0 (60.0–80.0)	0.04	58.0 (50.0–74.0)	70.0 (60.0–80.0)	0.22
Biomarkers												
CRP (mg/L)^a^	3.1 (2.0–5.7)	3.9 (1.9–8.0)	0.38	2.7 (1.8–3.8)	3.3 (1.7–5.3)	0.42	2.4 (1.7–4.4)	3.1 (1.8–5.0)	0.43	2.5 (1.8–3.9)	2.8 (1.7–4.9)	0.48
Triglycerides (nmol/L)^a^	2.9 (2.3–3.6)	2.4 (1.9–3.3)	0.08	2.8 (2.4–3.3)	2.3 (1.9–3.2)	0.16	3.0 (2.3–3.4)	2.2 (1.9–3.4)	0.06	2.8 (2.4–3.3)	2.1 (1.9–3.3)	0.23
Cholesterol (nmol/L)^a^	6.0 (5.4–7.3)	6.3 (5.3–7.3)	0.91	6.6 (5.3–8.0)	5.8 (5.1–6.5)	0.13	6.4 (5.4–7.4)	6.0 (5.1–7.2)	0.67	6.6 (5.1–8.0)	5.8 (5.1–6.3)	0.17
Adiponectin (ng/mL)^a^	454.1 (343.0–535.8)	429.9 (314.3–586.9)	0.77	497.7 (438.6–541.2)	429.9 (329.4–587.9)	0.37	458.8 (340.4–544.4)	440.6 (313.9–589.8)	0.83	468.4 (438.6–541.2)	433.9 (314.3–587.9)	0.66
Leptin (ng/mL)^a^	2.2 (1.2–3.6)	2.1 (1.1–3.7)	0.79	1.7 (1.1–2.8)	1.8 (0.8–2.9)	0.95	2.1 (1.1–3.3)	1.8 (0.8–2.7)	0.41	1.6 (1.1–2.6)	1.7 (0.8–2.7)	0.84

Values are number (%) unless otherwise indicated.

*OGTT* oral glucose tolerance test.

^a^Values are medians (IQR).

^b^Among women with previous pregnancy >20 weeks’ gestation.

Fasting glycemic targets were achieved ≥80% of the time in the tighter target group by 35.9% (14/39) women compared with 62.8% (27/43) of women in the less tight group; similarly 61.5% (24/39) women achieved their postprandial targets ≥80% of the time compared with 65.1% (28/43) in the less tight group, and 28.2% (11/39) achieved both tighter fasting and postprandial targets ≥80% of the time compared to 48.8% (21/43) in the less tight group (Fig. 1).

### Maternal cardiometabolic and inflammatory markers at 36 weeks’ gestation

When comparing women who were allocated to use the tighter targets to women allocated to use the less tight targets, there were no differences in maternal adiponectin, leptin, CRP, and triglyceride concentrations (Table 2). There was a −32.7% difference in cholesterol concentrations, ranging from −56.4% to 3.8% (*p* = 0.08), in women randomized to tighter glycemic targets when compared to women randomized to less tight targets. These results may indicate a clinically important difference in cholesterol concentrations.

**Table 2:** Maternal and neonatal cardiometabolic and inflammatory markers across pregnancy and postpartum between the study groups.

Outcomes	Number (tighter/less tight)	Tighter targets	Less tight targets	Unadjusted mean difference in percentage (95% CI)	Unadjusted *P* value	Adjusted^a^ mean difference in percentage (95% CI)	Adjusted^a^ *P* value	Adjusted^b^ mean difference in percentage (95% CI)	Adjusted^b^ *P* value
CRP (mg/L)									
Trial entry	39/43	3.7 (2.2)	6.4 (7.4)	−37.6 (−59.9, −2.9)	0.04	−36.9 (−59.4, −1.7)	0.05	−24.5 (−51.8, 18.1)	0.22
36 weeks	38/43	4.5 (3.5)	5.0 (3.5)	−33.4 (−56.7, 2.5)	0.07	−9.9 (−35.2, 25.2)	0.54	−5.0 (−32.8, 34.3)	0.77
6 months	39/43	3.0 (2.9)	4.3 (6.1)	−2.5 (−49.6, 88.7)	0.94	43.9 (−16.9, 149.1)	0.20	55.7 (−9.0, 166.4)	0.11
Adiponectin (ng/mL)									
Trial entry	39/43	456.8 (156.2)	470.2 (210.3)	9.5 (−10.7, 34.4)	0.39	9.7 (−10.7, 34.7)	0.38	11.7 (−8.9, 37.0)	0.29
36 weeks	38/43	504.9 (175.0)	511.8 (214.2)	8.1 (−12.0, 32.7)	0.46	−0.3 (−10.6, 11.1)	0.95	0.6 (−10.8, 13.4)	0.92
6 months	39/43	490.8 (216.1)	507.4 (231.0)	2.4 (−18.9, 29.3)	0.84	−3.9 (−19.1,14.2)	0.65	−9.7 (−24.1, 7.4)	0.25
Leptin (ng/mL)									
Trial entry	39/43	2.7 (2.3)	2.5 (1.8)	−12.9 (−43.1, 33.4)	0.53	−13.6 (−43.6, 32.4)	0.50	19.0 (−15.0, 66.5)	0.31
36 weeks	38/43	2.8 (2.5)	2.6 (1.5)	−23.8 (−49.3, 14.6)	0.20	−12.8 (−27.6, 4.9)	0.15	−9.7 (−26.2, 10.4)	0.32
6 months	39/43	2.2 (1.8)	1.9 (1.4)	−19.3 (−50.1, 30.5)	0.38	−7.7 (−32.1, 25.3)	0.61	−9.4 (−33.9, 24.1)	0.54
Triglycerides (nmol/L)									
Trial entry	39/43	3.0 (1.1)	2.7 (1.0)	16.2 (−3.7, 40.1)	0.12	14.7 (−4.8, 38.3)	0.15	18.7 (−2.9, 45.0)	0.10
36 weeks	38/43	3.6 (1.4)	3.3 (1.5)	8.5 (−11.5, 32.9)	0.44	−3.9 (−15.5, 9.3)	0.55	−0.7 (−13.7, 14.2)	0.92
6 months	39/43	1.4 (0.8)	1.4 (0.8)	−5.7 (−30.3, 27.4)	0.70	−18.6 (−36.7, 4.7)	0.11	−5.8 (−27.3, 22.1)	0.65
Cholesterol (nmol/L)									
Trial entry	39/43	6.3 (1.4)	6.3 (1.3)	4.7 (−7.3, 18.1)	0.46	3.7 (−8.1, 16.9)	0.56	3.9 (−7.8, 17.2)	0.53
36 weeks	38/43	6.4 (1.4)	6.5 (1.3)	−33.4 (−56.7, 2.5)	0.07	−32.7 (−56.4, 3.8)	0.08	−19.3 (−48.9, 27.3)	0.36
6 months	39/43	4.9 (0.8)	5.1 (1.0)	−2.5 (−49.6, 88.7)	0.94	−1.7 (−49.0, 89.4)	0.96	36.6 (−25.4, 150.3)	0.32
Adiponectin (ng/mL)									
Cord plasma	39/43	2049.6 (788.0)	1867.7 (772.9)	12.6 (−11.4, 43.0)	0.33	12.0 (−11.9, 42.4)	0.36	9.5 (−14.9, 40.9)	0.48
Leptin (ng/mL)									
Cord blood	39/43	1.8 (1.8)	1.9 (2.6)	−16.7 (−50.2, 39.6)	0.49	−17.7 (−51.0, 38.1)	0.46	−16.0 (−50.9, 43.6)	0.53
C peptide (ng/mL)									
Cord plasma	20/31	1.4 (0.8)	1.7 (0.8)	−21.8 (−47.9, 17.4)	0.24	−24.6 (−49.9, 13.5)	0.18	−15.1 (−45.3, 31.7)	0.47
IGF (ng/mL)									
Cord plasma	39/43	84.9 (38.7)	68.6 (32.3)	22.0 (−14.4, 74.1)	0.28	19.6 (−16.1, 70.6)	0.33	8.7 (−26.0, 59.8)	0.67

Data are presented as mean (SD), and the treatment effects are differences in percentage between the mean of the tighter and the less tight glycemic target group (95% CI), estimated from linear mixed-effects model, with random effects for hospital groups and women, and fixed effects for the intervention and time interval between the assigned targets initiated and a woman recruited, unless otherwise indicated.

^a^Adjusted for gestational age at OGTT (weeks) and baseline values.

^b^Adjusted for GA at OGTT (weeks), baseline values, ethnicity, BMI, and history of GDM.

There were no differences in serum CRP, adiponectin, triglyceride, and cholesterol concentrations for women who achieved at least 80% adherence with fasting targets, postprandial targets, or both fasting and postprandial targets between the two treatment groups (Tables 3–5).

**Table 3:** Fasting compliance with at least 80% of all fasting PG readings within targets—maternal and infant cardiometabolic and inflammatory markers.

Outcomes	Number (tighter/less tight)	Tighter targets	Less tight targets	Unadjusted mean difference in percentage (95% CI)	Unadjusted *P* value	Adjusted^a^ mean difference in percentage (95% CI)	Adjusted^a^ *P* value	Adjusted^b^ mean difference in percentage (95% CI)	Adjusted^b^ *P* value
CRP (mg/L)									
Trial entry	14/27	2.88 (1.38)	4.71 (4.54)	−26.90 (−65.41, 54.48)	0.42	−27.14 (−65.63, 54.42)	0.41	−24.32 (−63.77, 58.07)	0.46
36 weeks	13/27	5.08 (4.63)	4.02 (2.78)	−23.15 (−65.59, 71.61)	0.52	−6.67 (−51.14, 78.28)	0.84	2.74 (−48.10, 103.39)	0.94
6 months	14/27	1.40 (1.04)	2.69 (2.99)	7.89 (−63.93, 222.71)	0.89	44.53 (−41.32, 255.97)	0.43	63.87 (−27.54, 270.62)	0.25
Adiponectin (ng/mL)									
Trial entry	14/27	498.46 (156.69)	487.86 (233.50)	24.16 (−13.16, 77.51)	0.24	27.18 (−10.32, 80.38)	0.19	15.67 (−15.44, 58.22)	0.37
36 weeks	13/27	5244.17 (162.08)	526.19 (224.15)	12.31 (−20.75, 59.17)	0.52	−7.14 (−25.71, 16.09)	0.52	−9.38 (−27.98, 14.02)	0.41
6 months	14/27	584.96 (245.04)	527.47 (237.26)	13.44 (−23.64, 68.53)	0.54	−0.87 (−28.30, 37.05)	0.96	−9.09 (−33.86, 24.95)	0.56
Leptin (ng/mL)									
Trial entry	14/27	1.97 (1.24)	2.10 (1.41)	−24.59 (−63.64, 56.40)	0.45	−25.50 (−64.14, 54.78)	0.44	−4.81 (−44.18, 62.33)	0.86
36 weeks	13/27	1.82 (1.37)	2.41 (1.70)	−50.33 (−76.17, 3.51)	0.07	−39.22 (−54.72, −18.42)	0.002	−36.28 (−52.72, −14.11)	0.01
6 months	14/27	1.61 (1.09)	1.72 (1.29)	−45.11 (−76.60, 28.79)	0.18	−29.33 (−58.01, 18.92)	0.20	−33.85 (−59.97, 9.31)	0.12
Triglycerides (nmol/L)									
Trial entry	14/27	2.76 (0.78)	2.41 (0.71)	31.15 (−1.57, 74.75)	0.07	29.45 (−2.63, 72.09)	0.08	28.20 (−4.83, 72.69)	0.11
36 weeks	13/27	3.16 (0.76)	3.08 (1.57)	16.86 (−18.32, 67.19)	0.40	−6.08 (−28.14, 22.75)	0.65	−8.10 (−30.53, 21.57)	0.56
6 months	14/27	1.20 (0.72)	1.32 (0.78)	3.76 (−39.03, 76.57)	0.89	−25.27 (−51.04, 14.06)	0.19	−13.14 (−42.21, 30.56)	0.50
Cholesterol (nmol/L)									
Trial entry	14/27	6.59 (1.35)	5.94 (1.29)	30.18 (7.27, 57.98)	0.01	28.38 (6.33, 55.00)	0.01	20.44 (0.11, 44.89)	0.06
36 weeks	13/27	6.28 (1.35)	6.30 (1.30)	−23.15 (−65.59, 71.61)	0.52	−20.20 (−66.21, 88.47)	0.61	−12.90 (−63.22, 106.25)	0.76
6 months	14/27	4.81 (0.73)	4.90 (0.98)	7.89 (−63.93, 222.71)	0.89	40.41 (−56.32, 351.41)	0.57	39.95 (−47.79, 275.08)	0.51
Cord adiponectin (ng/mL)									
Cord plasma	14/27	1941.80 (807.83)	1870.09 (774.91)	−1.06 (−34.73, 49.97)	0.96	−0.59 (−34.50, 50.89)	0.98	−11.95 (−39.44, 28.02)	0.51
Cord leptin (ng/mL)									
Cord plasma	14/27	1.21 (0.93)	2.16 (3.14)	−56.25 (−82.44, 9.00)	0.08	−56.88 (−82.73, 7.68)	0.08	−58.63 (−83.53, 3.91)	0.07
Cord C peptide (ng/mL)									
Cord plasma	9/12	1.25 (0.70)	2.01 (0.83)	−39.41 (−64.87, 4.53)	0.09	−39.92 (−64.74, 2.37)	0.08	−49.04 (−66.27, −22.99)	0.01
Cord IGF (ng/mL)									
Cord plasma	14/26	72.66 (44.14)	64.85 (35.29)	−15.36 (−59.44, 76.62)	0.66	−18.67 (−60.62, 67.99)	0.58	−31.99 (−67.86, 43.90)	0.32

Data presented as mean (SD), and the treatment effects are difference in percentage between the mean of the tighter and the less tight glycemic target group (95% CI), estimated from linear mixed-effects model, with random effects for hospital groups and women, and fixed effects for the intervention and time interval between the assigned targets initiated and a woman recruited, unless otherwise indicated.

^a^Adjusted for gestational age at OGTT (weeks) and baseline values.

^b^Adjusted for GA at OGTT (weeks), baseline values, ethnicity, BMI, and history of GDM.

**Table 4:** Postprandial compliance with at least 80% of all postprandial PG readings within targets—maternal and infant cardiometabolic and inflammatory markers.

Outcomes	Number (tighter/less tight)	Tighter targets	Less tight targets	Unadjusted mean difference in percentage (95% CI)	Unadjusted *P* value	Adjusted^a^ mean difference in percentage (95% CI)	Adjusted^a^ *P* value	Adjusted^b^ mean difference in percentage (95% CI)	Adjusted^b^ *P* value
CRP (mg/L)									
Trial entry	24/28	3.25 (2.03)	5.54 (7.25)	−29.58 (−58.50, 19.50)	0.20	−26.20 (−56.52, 25.28)	0.27	−20.80 (−53.73, 35.57)	0.40
36 weeks	23/28	4.38 (3.96)	4.41 (3.34)	−19.89 (−53.69, 38.59)	0.43	1.86 (−31.64, 51.78)	0.93	6.59 (−30.98, 64.62)	0.78
6 months	24/28	2.40 (2.56)	2.57 (2.73)	35.63 (−36.63, 190.30)	0.44	74.35 (−7.58, 228.89)	0.09	122.80 (21.22, 309.51)	0.01
Adiponectin (ng/mL)									
Trial entry	24/28	452.22 (147.86)	496.82 (236.66)	5.00 (−19.00, 36.11)	0.71	4.02 (−19.97, 35.20)	0.77	6.20 (−16.95, 35.80)	0.63
36 weeks	23/28	494.10 (175.09)	529.29 (228.48)	6.38 (−17.24, 36.75)	0.63	1.88 (−11.85, 17.75)	0.80	6.31 (−9.65, 25.10)	0.47
6 months	24/28	479.41 (189.83)	545.46 (264.37)	−4.53 (−28.77, 27.96)	0.76	−7.40 (−25.45, 15.03)	0.49	−9.58 (−27.77, 13.19)	0.38
Leptin (ng/mL)									
Trial entry	24/28	2.26 (1.29)	2.09 (1.63)	2.59 (−36.93, 66.88)	0.92	5.48 (−35.39, 72.21)	0.83	30.46 (−12.43, 94.36)	0.20
36 weeks	23/28	2.38 (1.54)	2.35 (1.58)	−14.54 (−45.63, 34.31)	0.50	−17.82 (−34.39, 2.94)	0.09	−19.90 (−38.11, 3.68)	0.10
6 months	24/28	2.00 (1.53)	1.74 (1.37)	−6.26 (−49.04, 72.41)	0.84	−13.06 (−40.69, 27.45)	0.48	−15.23 (−43.55, 27.28)	0.43
Triglycerides (nmol/L)									
Trial entry	24/28	3.02 (1.11)	2.62 (1.00)	31.34 (5.68, 63.24)	0.02	29.54 (4.08, 61.21)	0.02	34.62 (5.77, 71.34)	0.02
36 weeks	23/28	3.36 (1.43)	3.16 (1.25)	16.44 (−7.97, 47.31)	0.21	−7.95 (−18.86, 4.43)	0.20	−5.26 (−17.58, 8.90)	0.45
6 months	24/28	1.25 (0.59)	1.32 (0.72)	3.32 (−25.74, 43.76)	0.85	−17.82 (−38.76, 10.27)	0.20	−3.18 (−28.50, 31.10)	0.84
Cholesterol (nmol/L)									
Trial entry	24/28	6.38 (1.47)	6.14 (1.44)	13.21 (−2.76, 31.80)	0.12	11.67 (−4.08, 30.01)	0.16	9.96 (−5.47, 27.90)	0.23
36 weeks	23/28	6.41 (1.44)	6.40 (1.34)	−19.89 (−53.69, 38.59)	0.43	−15.36 (−51.68, 48.24)	0.56	−10.51 (−50.20, 60.81)	0.71
6 months	24/28	4.92 (0.82)	5.03 (1.16)	35.63 (−36.63, 190.30)	0.44	54.84 (−27.65, 231.36)	0.27	98.33 (−0.54, 295.51)	0.06
Cord adiponectin (ng/mL)									
Cord blood	24/28	1872.86 (763.44)	1944.44 (826.79)	1.75 (−24.78, 37.65)	0.91	1.12 (−25.51, 37.29)	0.94	3.46 (−24.75, 42.25)	0.84
Cord leptin (ng/mL)									
Cord blood	24/28	1.96 (2.03)	2.16 (3.09)	−8.36 (−52.24, 75.82)	0.79	−5.23 (−50.88, 82.84)	0.87	−11.46 (−56.10, 78.54)	0.74
Cord C peptide (ng/mL)									
Cord blood	14/14	1.32 (0.72)	1.75 (0.95)	−18.24 (−50.39, 34.72)	0.44	−23.24 (−54.26, 28.81)	0.33	−13.81 (−51.59, 53.43)	0.62
Cord IGF (ng/mL)									
Cord blood	24/27	94.85 (41.95)	59.17 (28.54)	66.47 (4.65, 164.80)	0.04	69.14 (5.80, 170.40)	0.03	31.28 (−20.79, 117.57)	0.30

Data presented as mean (SD), and the treatment effects are difference in percentage between the mean of the tighter and the less tight glycemic target group (95% CI), estimated from linear mixed-effects model, with random effects for hospital groups and women, and fixed effects for the intervention and time interval between the assigned targets initiated and a woman recruited, unless otherwise indicated.

^a^Adjusted for gestational age at OGTT (weeks) and baseline values.

^b^Adjusted for GA at OGTT (weeks), baseline values, ethnicity, BMI, and history of GDM.

**Table 5:** Both fasting and postprandial compliance with at least 80% of all fasting and postprandial PG readings within targets—maternal and infant cardiometabolic and inflammatory markers.

Outcomes	Number (tighter/less tight)	Tighter targets	Less tight targets	Unadjusted mean difference in percentage (95% CI)	Unadjusted *P* value	Adjusted^a^ mean difference in percentage (95% CI)	Adjusted^a^ *P* value	Adjusted^b^ mean difference in percentage (95% CI)	Adjusted^b^ *P* value
CRP (mg/L)									
Trial entry	11/21	2.88 (1.56)	4.03 (3.46)	−25.97 (−62.70, 46.96)	0.40	−27.69 (−63.60, 43.64)	0.36	−23.30 (−61.37, 52.30)	0.46
36 weeks	10/21	5.22 (5.22)	4.06 (2.87)	−19.57 (−66.26, 91.76)	0.63	−0.39 (−50.03, 98.55)	0.99	−1.16 (−51.86, 102.94)	0.98
6 months	11/21	1.33 (0.81)	2.33 (2.39)	5.69 (−64.16, 211.70)	0.92	37.12 (−47.00, 254.75)	0.52	61.82 (−32.20, 286.23)	0.29
Adiponectin (ng/mL)									
Trial entry	11/21	470.74 (100.92)	497.04 (254.61)	23.22 (−14.87, 78.36)	0.28	26.47 (−12.10, 81.96)	0.22	24.79 (−8.75, 70.66)	0.18
36 weeks	10/21	481.66 (110.15)	516.54 (224.77)	11.68 (−20.48, 56.84)	0.53	−4.25 (−24.34, 21.19)	0.72	−2.30 (−22.32, 22.88)	0.84
6 months	11/21	540.29 (181.32)	536.93 (260.28)	10.98 (−26.64, 67.89)	0.63	−1.36 (−30.89, 40.80)	0.94	−6.23 (−34.09, 33.39)	0.72
Leptin (ng/mL)									
Trial entry	11/21	1.97 (1.23)	1.92 (1.31)	−12.48 (−54.36, 67.81)	0.69	−11.01 (−53.70, 71.03)	0.73	−7.05 (−45.94, 59.80)	0.79
36 weeks	10/21	1.87 (1.43)	2.29 (1.66)	−44.65 (−71.47, 7.41)	0.09	−41.33 (−57.36, −19.28)	0.003	−37.14 (−54.24, −13.66)	0.01
6 months	11/21	1.53 (1.07)	1.56 (1.00)	−40.60 (−73.68, 34.07)	0.22	−33.30 (−60.28, 12.03)	0.14	−29.13 (−57.04, 16.92)	0.19
Triglycerides (nmol/L)									
Trial entry	11/21	2.80 (0.70)	2.45 (0.77)	23.98 (−7.89, 66.89)	0.17	22.82 (−8.81, 65.42)	0.19	25.20 (−7.93, 70.24)	0.17
36 weeks	10/21	3.16 (0.66)	2.92 (0.97)	13.94 (−16.77, 55.98)	0.42	−1.56 (−19.90, 20.99)	0.88	−2.36 (−21.03, 20.74)	0.83
6 months	11/21	1.14 (0.72)	1.33 (0.79)	−0.87 (−42.41, 70.65)	0.98	−23.71 (−50.21, 16.90)	0.23	−4.14 (−33.10, 37.34)	0.82
Cholesterol (nmol/L)									
Trial entry	11/21	6.57 (1.39)	5.90 (1.38)	28.39 (4.15, 58.29)	0.03	26.94 (3.12, 56.27)	0.03	22.00 (−0.30, 49.29)	0.07
36 weeks	10/21	6.14 (1.24)	6.22 (1.29)	−19.57 (−66.26, 91.76)	0.63	−23.77 (−69.89, 92.99)	0.57	−17.00 (−66.92, 108.27)	0.70
6 months	11/21	4.75 (0.77)	4.99 (1.03)	5.69 (−64.16, 211.70)	0.92	35.13 (−57.15, 326.16)	0.61	41.00 (−50.79, 304.00)	0.53
Cord adiponectin (ng/mL)									
Cord blood	11/21	1870.14 (849.75)	2013.04 (790.49)	−10.06 (−41.24, 37.65)	0.63	−8.22 (−39.94, 40.24)	0.69	−9.85 (−39.45, 34.20)	0.61
Cord leptin (ng/mL)									
Cord blood	11/21	1.13 (0.93)	2.45 (3.52)	−54.90 (−83.41, 22.62)	0.13	−54.81 (−83.49, 23.66)	0.13	−62.57 (−85.97, 0.16)	0.06
Cord C peptide (ng/mL)									
Cord plasma	7/10	1.25 (0.79)	2.12 (0.86)	−44.20 (−69.05, 0.58)	0.08	−44.01 (−68.79, 0.42)	0.08	−54.00 (−65.79, −38.14)	0.001
Cord IGF (ng/mL)									
Cord plasma	11/20	72.20 (46.72)	59.00 (31.32)	−9.06 (−58.47, 99.14)	0.81	−10.37 (−59.23, 97.01)	0.79	−31.74 (−67.88, 45.06)	0.33

Data presented as mean (SD), and the treatment effects are difference in percentage between the mean of the tighter and the less tight glycemic target group (95% CI), estimated from linear mixed-effects model, with random effects for hospital groups and women, and fixed effects for the intervention and time interval between the assigned targets initiated and a woman recruited, unless otherwise indicated.

^a^Adjusted for gestational age at OGTT (weeks) and baseline values.

^b^Adjusted for GA at OGTT (weeks), baseline values, ethnicity, BMI, and history of GDM.

Women who achieved ≥80% of tighter fasting glycemic targets had lower serum leptin concentrations compared to women who achieved ≥80% of less tight fasting glycemic targets after adjustment for GA at OGTT and baseline values (mean difference −39.2%, 95% CI −54.7 to −18.4, *P* = 0.002). This decrease remained significant after further adjustments for ethnicity, BMI, and history of GDM (−36.3% difference, 95% CI −52.7 to −14.1, *P* = 0.006).

There was a reduction in maternal serum leptin concentrations at 36 weeks’ gestation in women who achieved ≥80% of both tighter fasting and postprandial targets after adjustment for GA at OGTT and baseline values (−41.3% decrease, 95% CI −57.4 to −19.3, *P* = 0.003). This decrease remained significant after adjustment for ethnicity, BMI, and history of GDM (−37.1% decrease, 95% CI −54.2 to −13.7, *P* = 0.01).

### Maternal cardiometabolic and inflammatory markers at 6 months postpartum

When comparing women who used the tighter targets to women who used the less tight targets, there were no differences in maternal adiponectin, leptin, and triglyceride concentrations at 6 months postpartum (Table 2). Women randomized to tighter glycemic targets experienced a 55.7% difference in CRP concentrations ranging from −9.03% to 166.36% after adjustment for GA at OGTT, baseline values, ethnicity, BMI, and history of GDM. This greater than 50% difference may indicate a clinically relevant change in CRP concentrations when randomized to tighter glycemic targets.

There were no differences at 6 months found in serum adiponectin, triglyceride, and leptin concentrations for women who achieved at least 80% adherence with fasting targets, postprandial targets or both fasting and postprandial targets between the two treatment groups (Tables 3–5).

At 6 months postpartum in women who achieved ≥80% of postprandial targets in the tighter glycemic targets group, there was a 122.8% (95% CI 21.22 to 309.51, *P* = 0.01) increase in CRP concentrations, compared with those in the less tight glycemic targets group after adjustment for GA at OGTT, baseline values, ethnicity, BMI and history of GDM. There was also a 98.3% increase in cholesterol concentration, ranging from −0.5 to 295.5 (*P* = 0.06), in women allocated to tighter glycemic targets at 6 months postpartum who achieved ≥80% of postprandial targets compared to women who were allocated to less tight glycemic targets and achieved ≥80% of postprandial targets.

### Neonatal cardiometabolic, growth, and inflammatory markers

There was no difference in adiponectin, leptin, and IGF concentrations in cord plasma when comparing infants of women using tighter targets with those using less tight targets (Table 2). There was a −24.6% reduction (95% CI −49.9 to 13.5) in C-peptide concentration in infants of mothers randomized to tighter glycemic targets compared to infants of mothers randomized to less tight glycemic targets, a reduction that may be of clinical relevance.

Cord plasma adiponectin concentrations in infants of mothers who achieved at least 80% adherence with fasting targets, postprandial targets, or both fasting and postprandial targets did not differ between the two treatment groups (Tables 3–5).

Cord C-peptide concentrations were lower in infants of women who achieved ≥80% adherence to the tighter fasting glycemic targets compared with infants of women who achieved ≥80% adherence to the less tight fasting glycemic targets after adjustment for GA at OGTT, baseline values, ethnicity, BMI, and history of GDM (mean difference −49.0%, 95% CI −66.3 to −23.0, *P* = 0.01; Table 3).

In infants of women who achieved ≥80% of tighter postprandial targets, there was a 66.5% increase in unadjusted analyses (95% CI 4.7 to 164.8, *P* value 0.04) in cord plasma IGF concentrations compared with those who achieved ≥80% of less tight postprandial targets. After adjustment for GA at OGTT and baseline values were made, the 69.1% increase remained significant (95% CI 5.8 to 170.5, *P* = 0.03). However, after further adjustment for ethnicity, BMI, and history of GDM, there was no longer a significant difference between the two groups (31.3%, 95% CI −20.8. to 117.6, *P* = 0.30).

When both fasting and postprandial targets could be achieved, infants of women in the tighter glycemic target group had a −54.0% decrease, ranging from −65.8 to −38.1 (*P* = 0.001), in cord C-peptide concentrations compared to infants of women who achieved ≥80% of plasma readings in the less tight glycemic target group.

Infants of women who achieved both tighter fasting and postprandial glycemic targets ≥80% of the time had a −62.57% decrease in cord leptin concentration, ranging from −85.97 to 0.16 (*P* = 0.06), after adjustment for GA at OGTT, baseline values, ethnicity, BMI, and history of GDM. The large treatment size of a 63% decrease of cord leptin concentration among infants whose mother were in the tighter target group with the marginally statistical significance (*P* = 0.06) may be of clinical importance.

## Discussion

Overall, the results of our study suggest that the use of tighter glycemic targets, as used in the TARGET Trial, do not result in an alteration in maternal or infant cord plasma cytokine and adipokine concentrations when compared to women who were using less tight glycemic targets. Although the results do suggest there may be clinically important changes in maternal CRP and cholesterol concentrations and infant cord plasma C-peptide concentrations.

Women using tighter glycemic targets who achieved ≥80% compliance with fasting, postprandial, or both fasting and postprandial glycemic targets had an altered maternal serum and cord plasma biomarker profile compared to women using less tight glycemic targets who achieved ≥80% compliance. Women who achieved compliance with tighter fasting glycemic targets had lower maternal leptin concentrations at 36 weeks’ gestation when adjusted for potential confounding factors, and their infants had lower C-peptide cord plasma concentrations compared with women who achieved compliance with the less tight glycemic targets. At 6 months after the birth when postprandial targets were achieved in the tighter targets group, women had a raised CRP and cholesterol concentrations and cord IGF concentrations were increased in their infants compared to those who achieved less tight postprandial targets. Women who achieved both fasting and postprandial targets in the tighter targets group had lower maternal leptin concentrations at 36 weeks’ gestation when adjusted for potential confounding factors. Infants of women achieving both tighter fasting and postprandial glycemic targets experienced a decrease in cord plasma C-peptide concentrations and there was a likely decrease in cord plasma leptin concentrations compared to those achieving less tight glycemic targets. These results indicate that significant changes do occur in maternal and infant biomarkers when high compliance with tighter glycemic targets can be achieved. The results of our study further contribute to the growing literature base surrounding the benefits and implications of tighter glycemic control in the management of gestational diabetes mellitus.

A meta-analysis^4^ identified a correlation between GDM and raised serum leptin and suggested that high maternal leptin may contribute to insulin resistance. Our results suggest that serum leptin concentrations are reduced when women achieve high compliance with tighter glycemic targets, particularly with fasting blood glucose concentrations. It is important to consider the impact a reduction in serum leptin may have on insulin resistance in GDM and, therefore, a potential reduction in the short-term and long-term consequences of insulin resistance. However, our results at 6 months postpartum suggest no long-term difference in leptin concentrations between women who used tight glycemic control and less tight glycemic control, suggesting no long-term effect of the reduced serum leptin at 36 weeks’ gestation, even if compliance with glycemic control has been good.

Several previous analyses have reported raised maternal serum CRP in women with GDM, particularly in the third trimester, compared with women without GDM.^5–7^ One report showed a significant correlation between CRP concentration and insulin and blood glucose concentrations.^7^ Women in our study who achieved high compliance to tighter postprandial glycemic targets had an increase in CRP concentrations at 6 months postpartum. This result contrasts with what would be anticipated given the association between blood glucose concentrations and CRP concentrations. The small sample size may have limited our ability to detect differences, and there is a need for further research assessing inflammatory markers and glycemic control of GDM.

A systematic review of observational studies identified that maternal adiponectin concentrations were decreased in GDM compared with women without GDM.^4^ Our results suggest that although adiponectin concentrations may decrease in GDM, tighter treatment targets did not lead to a different effect compared to the use of less tight targets. This may be because blood glucose concentrations alone do not cause the changes in serum adiponectin.

Although overall no difference was found in cord plasma leptin concentration between the glycemic target groups, reduced cord plasma leptin in infants of women who complied with tighter glycemic targets after adjustment for potential confounding factors is consistent with the results from other studies.^18,19^ A nested study within the ACHOIS Trial showed a decrease in cord plasma leptin concentrations when comparing women with mild GDM who received dietary advice and pharmacological treatment when required to women with mild GDM who received routine pregnancy care.^18^ A further small trial that randomized women to tight glycemic targets and less tight glycemic targets identified reduced cord leptin concentrations in those assigned to tight glycemic targets.^19^ Cord plasma leptin is correlated with neonatal adiposity, a significant risk factor for childhood obesity.^12^ Therefore, a reduction in leptin associated with tighter glycemic control may be important for preventing neonatal adiposity and subsequent complications of childhood obesity and metabolic syndrome.^29^

The nested study within the ACHOIS Trial showed a decrease in cord plasma adiponectin in infants of women diagnosed with GDM, although there was no difference between infants of women treated for their GDM and infants of women who did not receive treatment.^18^ Our study results and also those from another randomized trial, that included 41 women randomized to tight glycemic targets (fasting blood glucose <5.1 mmol/L and <7.0 mmol/L postprandial) and less tight glycemic targets (<5.3 mmol/L and <7.8 mmol/L, respectively) and 25 women without GDM as controls, confirm these earlier results that tighter glycemic control did not seem to influence cord adiponectin concentrations.^19^

Our results suggest that tighter glycemic control results in reduced cord C-peptide concentrations. Several cohort studies have shown that cord C-peptide is raised in infants of women with GDM and suggest the insulin-secretory activity of an infant is related to maternal metabolic parameters, glycemic, and insulin homeostasis during pregnancy.^30,31^ The HAPO study identified that infants who developed hypoglycemia after birth were strongly associated with an elevated cord C-peptide concentration.^32^ Another study identified that C-peptide is positively correlated with birth weight.^33^ Therefore, a reduction in cord C-peptide concentration in infants of women who achieved tight glycemic control may highlight a clinically meaningful change that will likely impact other infant outcomes. In contrast, a randomized trial assessing tight and less tight glycemic targets in women with GDM reported no significant difference in cord C-peptide.^19^ Data on glycemic control from electronic diaries showed average fasting blood glucose values of 4.7 and 4.8 mmol/L and postprandial blood glucose values of 5.9 and 6.4 mmol/L in the tighter targets group and the less tight targets group, respectively. These average values suggest that relatively tight control was achieved in both groups. It is unclear why this study yielded different results to our current study, but it may be due to the different glycemic targets used, different assays that were used, or that both studies had limited sample sizes.

Previous analyses have described increased cord plasma IGF concentrations in infants of women with GDM.^34,35^ However, our results suggest that cord plasma IGF concentrations increase with tighter postprandial glycemic targets, are at variance with this. When the analyses were adjusted for ethnicity, BMI, and history of GDM, the increase in IGF was no longer statistically significant, suggesting that other factors beyond glycemic control may have contributed to this change in IGF concentration. These conflicting results highlight the need for further research into the impact of glycemic control on cord IGF.

There are several strengths to our study. These include the randomized controlled trial study design, which reduces the risk of confounding. By obtaining cardiometabolic marker concentrations during pregnancy, at birth, and postnatally, we were able to assess the impact of tight glycemic targets compared to less tight targets for women with GDM over time, not just around birth. In addition, we were better able to observe the true impact of optimal glycemic control on cardiometabolic markers by assessing compliance with glycemic control. Our study was limited by our sample size, particularly given the small number of women who achieved high compliance with the tighter targets and may have limited our ability to detect true differences in biomarker concentrations.

## Conclusion

In this study the overall use of tighter targets for glycemic control in women with GDM did not result in a difference in concentrations of cardiometabolic markers in maternal serum and infant cord plasma when compared to less tight targets. The use of tighter targets for glycemic control in women with GDM, who achieved ≥80% of fasting or both fasting and postprandial targets, resulted in a reduction in maternal serum leptin concentrations and infant cord C-peptide and leptin concentrations compared to similar high compliance to less tight targets. However, achieving tighter postprandial glycemic targets may increase cord IGF concentrations and 6 months after the birth maternal serum CRP and cholesterol concentrations. Further research is required to assess the clinical benefit of tighter glycemic control and the barriers and enablers to achieving high compliance to these targets.

## Data Availability

The datasets generated during and/or analyzed during the current study are available from the corresponding author on reasonable request.
